# Nomogram Predicting Lymph Node Metastasis in the Early-Stage Cervical Cancer

**DOI:** 10.3389/fmed.2022.866283

**Published:** 2022-06-30

**Authors:** Shimin Yang, Chunli Liu, Chunbo Li, Keqin Hua

**Affiliations:** ^1^Department of Obstetrics and Gynecology, Obstetrics and Gynecology Hospital, Fudan University, Shanghai, China; ^2^Shanghai Universal Medical Imaging Diagnostic Center, Shanghai, China

**Keywords:** cervical cancer, nomogram, lymph node metastasis, PET/CT, decision curve analysis

## Abstract

**Background:**

Accurately predicting the risk level of lymph node metastasis is essential for the treatment of patients with early cervical cancer. The purpose of this study is to construct a new nomogram based on 2-deoxy-2-fluorodeoxyglucose positron emission tomography/computed tomography (^18^F-FDG PET/CT) and clinical characteristics to assess early-stage cervical cancer patients’ risk of lymph node metastasis.

**Materials and Methods:**

From January 2019 to November 2020, the records of 234 patients with stage IA-IIA [International Federation of Gynecology and Obstetrics (FIGO) 2018] cervical cancer who had undergone PET/CT examination within 30 days before surgery were retrospectively reviewed. A nomogram to predict the risk of lymph node metastasis was constructed based on it. The nomogram was developed and validated by internal and external validation. The validation cohorts included 191 cervical cancer patients from December 2020 to October 2021.

**Results:**

Four factors [squamous cell carcinoma associated antigen (SCCA), maximum standardized uptake value of lymph node (nSUVmax), uterine corpus invasion in PET/CT and tumor size in PET/CT] were finally determined as the predictors of the nomogram. At the area under the receiver operating characteristic curve cohort was 0.926 in the primary and was 0.897 in the validation cohort. The calibration curve shows good agreement between the predicted probability and the actual probability. The decision curve analysis showed the clinical utility of the nomogram.

**Conclusion:**

We had established and verified a simple and effective nomogram, which can be used to predict the lymph node metastasis of cervical cancer patients before surgery.

## Introduction

Cervical cancer (CC) is one of the most common gynecological malignancies worldwide. It is estimated that an estimated 569,847 new cases and 311,365 related deaths were diagnosed each year. More than 85% of these cases occur in developing countries (1). The treatment of choice depended on cancer stage. The hallmark of a good staging system is the ability to define anatomical extent of disease and differentiate survival outcomes. Cancer staging is an evolving process that responds to developments in technology that improves diagnosis and treatment. Since 2018, the presence of pelvic or para-aortic lymph node metastases assigns the case to stage IIIC regardless of other findings for CC instead of clinical assessment of staging (2). Because information on the lymph node (LN) status is necessary to determine the treatment strategy, an accurate and effective preoperative diagnostic approach for lymph node (LN) metastases is urgently required.

Imaging techniques, including magnetic resonance imaging (MRI), computed tomography (CT), positron emission tomography (PET), positron emission tomography/computed tomography (PET/CT), positron emission tomography/magnetic resonance imaging (PET/MRI), and trans-vaginal ultrasound, can detect lymph node involvement with CC, facilitate determination of spread to the retroperitoneum. However, the sensitivity of these methods for detecting nodal metastasis varies from 60 to 88% (3–5). The role of PET/CT to detect lymph nodal metastasis has been studied in various centers and the results are unsatisfactory with a sensitivity of 48.6–82% (6, 7). Several studies have added radiomics for LN metastasis prediction in CC. In early phase, Kim et al. constructed a nomogram based on age, tumor size by MRI, LN metastasis on PET/CT, and their model could accurately identify patients at low risk of lymph node metastasis (LNM). Similarity, Liu et al. developed a non-invasive and convenient nomogram based on two parameters [squamous cell carcinoma associated antigen (SCCA), maximum standardized uptake value of lymph node (nSUVmax)] for preoperative identification of pelvic lymph node metastasis in early-stage CC and reported a high sensitivity and specificity compared to single parameter (6, 8). Both the nSUVmax of PET/CT and SCCA is very important to confirm the diagnose of CC and the positive lymph node. However, their nomogram just focused on the 2-deoxy-2-fluorodeoxyglucose (^18^F-FDG) uptake of lymph node (LN), ignoring the size of tumor and the spread of tumor. It is known that endometrial cancer is associated with the high risk of lymphatic metastasis. Previous studies also reported that patients with uterine invasion of CC exhibited high rates of pelvic lymph node metastasis, increased risk of distant metastasis, high rates of recurrence, and poor survival. Recently, Turan et al. reported that patients with uterine invasion had a higher rate of para-aortic lymph node metastasis than patients without uterine invasion (35 vs. 22.8%, *p* = 0.046) (9). Thus, we believed that uterine invasion and size defined by PET/CT is very important to evaluate the pelvic lymph node metastasis.

In this study, we aimed to explore a simple and effective predictive model based on four factors (SCCA, nSUVmax, uterine corpus invasion, and tumor size in PET/CT) for LN metastasis of clinically early-stage cervical cancer. We defined a low-risk group of patients who would least benefit from a surgical treatment and pointed out a guideline in the decision for the need for adjuvant radiotherapy.

## Materials and Methods

### Patients

In this retrospective study, we enrolled 425 cervical cancer patients who underwent surgery at the Obstetrics and Gynecology Hospital of Fudan University from January 2019 to October 2021. All patients were diagnosed with early-stage CC [IA1 with lymph-vascular space invasion (LVSI), IA2-IIA2]. A total of 234 patients from January 2019 to November 2020 and 191 patients from December 2020 to October 2021 was assigned into primary and external validation cohorts, respectively. All private information is strictly secret and only used for the purpose of this research. The clinical and pathological staging of cervical cancer is based on FIGO 2018 guidelines. The ethics committee of Obstetrics and Gynecology Hospital of Fudan University approved this study.

The inclusion criteria were as follows: (1) patients had undergone PET/CT in the 30 days before surgery; (2) patients underwent pelvic lymph node dissection; (3) patients had no chemotherapy or radiation before PET/CT examination. The exclusion criteria were as follows: (1) patients were lack of postoperative pathological report; (2) pathological report suggested that patients had cervical carcinoma *in situ*; (3) Patients undergone hysterectomy or pelvic lymph node dissection before PET/CT examination.

Patients performed pelvic lymph node dissection, and the bilateral common iliac lymph nodes as well as obviously enlarged lymph nodes were used for frozen section during the operation. Not all patients performed para-aortic lymph node dissection. Patient characteristics including age, FIGO stage, number of pregnancies, preoperative SCCA level, menopause, pathological LN metastasis, and PET/CT image date were obtained from the medical records.

### Positron Emission Tomography/Computed Tomography Examination

All patients received PET/CT with an integrated PET/CT scanner (Biograph-64, Siemens, Munich, Germany) within the 30 days before surgery. The patients fasted for at least 6 h before the PET/CT examination. The blood glucose level of patients was controlled at <10.0 mmol/L before 2-deoxy-2-fluorodeoxyglucose (^18^F-FDG) injection (2.9–5.6 MBq/kg body weight). Before scanning, patients were required to be quiet for approximately 1 h after urination. Images were included CT and PET scans from the base of the skull to the mid-thigh level. Then, the attenuation correction of CT data was used to reconstruct PET image data sets and the images were displayed on a workstation.

The most common regions of lymph node metastases for cervical cancer patients are pelvic lymph nodes, para-aortic lymph nodes, and inguinal lymph nodes. Two experienced nuclear medicine physicians analyzed and interpreted all PET/CT parameters independently, such as LN in PET/CT, LN diameter, and maximum standardized uptake value (SUVmax). They were blindly without knowledge of patient’s information, medical data, and pathological results. We reported the lymph node status and uterine corpus invasion in PET/CT scan (the invasion of tumor beyond the internal cervical orifice in PET/CT), and measured the maximum standardized uptake value of tumor (tSUVmax), maximum standardized uptake value of lymph node (nSUVmax), tumor size and LN diameter in PET/CT, and also calculated the value of nSUVmax/tSUVmax. The pathological results served as the gold standard for comparison with the PET/CT results. Pathological stage follows FIGO 2018 guidelines.

### Statistical Analysis

IBM SPSS (version 23.0; IBM, Inc., Chicago, IL, United States) and R (version 4.1.2^1^; The R Foundation for Statistical Computing, Vienna, Austria) were used for statistical analysis. Continuous variables were described as mean with standard deviation (SD), and categorical variables were described as frequencies with percentages. Univariate and multivariable analysis were performed, and used an odds ratio (OR) with a 95% confidence interval (CI) to estimate correlation strength. Predictors (*P* < 0.05) in univariate analysis were entered a multivariable regression analysis. A nomogram capable of predicting the risk of lymph node metastasis in cervical cancer patients was then developed based on the multivariable regression analysis.

The performance of the nomogram was assessed internally and externally by discrimination and calibration. The discriminative ability of the nomogram in predicting lymph node metastasis was evaluated by calculating the area under the receiver operating characteristics curve (AUC-ROC). The calibration of the model was performed by comparing the predicted and actual probability of lymph node metastasis. The internal verification of the nomogram used 1,000 bootstrap resample in the primary cohort, and the model was applied to the validation cohort for external validation. In addition, decision curve (DCA) was used to determine the clinical usefulness of the nomogram by calculating the net benefits at different threshold probabilities in the primary data set (10). We also analyzed the sensitivity, specificity, positive predictive value (PPV), negative predictive value (NPV), and accuracy of PET/CT alone and nomogram in each cohort. *P* < 0.05 was considered statistically significant.

## Results

The characteristics of the enrolled patients are shown in Table 1. All patients underwent pelvic lymph node dissection during operation. In the primary cohort, LN metastasis was pathologically confirmed after surgery in 61 cases and 173 cases had no LN metastasis. In a survey, 49 (80.3%) patients with positive LN metastasis in PET/CT and pathological results. The mean (SD) of preoperative SCCA was 1.9 (2.4) ng/ml in patients with negative LN metastasis and the positive patients was 8.8 (13.1) ng/ml. The mean (SD) of nSUVmax of positive or negative LN metastasis was 4.5 (4.5) and 0.7 (1.1). In a survey, 27 (44.3%) cases with LN metastasis had uterine corpus invasion in PET/CT scan. According to the pathological reports, there were 60 cases with LN metastasis and 131 cases without LN metastasis among the validation cohort. Among them, 105 (80.2%) PET/CT scan-negative patients had no LN metastasis, while 42 (70.0%) PET/CT scan-positive patients were confirmed LN metastasis.

**TABLE 1:** Patient characteristics and univariate analysis of the risk of lymph node metastasis.

Variable	Primary cohort (*n* = 234)			Validation cohort (*n* = 191)		
	Node negative	Node positive	*P*	Node negative	Node positive	*P*
	(*n* = 173)	(*n* = 61)		(*n* = 131)	(*n* = 60)	
Age (year), n (%)			0.346			0.008
<50	83 (48.0)	25 (41.0)		64 (48.9)	17 (28.3)	
≥50	90 (52.0)	36 (59.0)		67 (51.1)	43 (71.7)	
Menopause, n (%)			0.257			0.027
No	94 (54.3)	28 (45.9)		75 (57.3)	24 (40.0)	
Yes	79 (45.7)	33 (54.1)		56 (42.7)	36 (60.0)	
Number of pregnancies, n (%)			0.442			0.635
<3	75 (43.4)	23 (37.7)		52 (39.7)	26 (43.3)	
≥3	98 (56.6)	38 (62.3)		79 (60.3)	34 (56.7)	
Hypertension, n (%)			0.185			0.500
No	151 (87.3)	49 (80.3)		112 (85.5)	49 (81.7)	
Yes	22 (12.7)	12 (19.7)		19 (14.5)	11 (18.3)	
Diabetes, n (%)			>1.000			>1.000
No	167 (96.5)	59 (96.7)		122 (93.1)	56 (93.3)	
Yes	6 (3.5)	2 (3.3)		9 (6.9)	4 (6.7)	
Tumor histology, n (%)			0.099			0.337
Squamous cell cancer	138 (79.8)	54 (88.6)		108 (82.4)	52 (86.7)	
Adenocarcinoma	22 (12.7)	6 (9.8)		13 (10.0)	2 (3.3)	
Adenosequamous cancer	9 (5.2)	0 (0.0)		7 (5.3)	5 (8.3)	
Others	4 (2.3)	1 (1.6)		3 (2.3)	1 (1.7)	
2018 FIGO stage, n (%)			<0.001			<0.001
IA	9 (5.2)	0 (0.0)		9 (6.9)	0 (0.0)	
IB	136 (78.6)	27 (44.3)		77 (58.8)	26 (43.3)	
IIA	28 (16.2)	34 (55.7)		45 (34.3)	34 (56.7)	
**SCCA (ng/mL)**						
Mean (SD)	1.9 (2.4)	8.8 (13.1)	<0.001	2.8 (3.4)	12.5 (15.3)	<0.001
**Tumor size in PET/CT (cm)**						
Mean (SD)	2.2 (1.7)	4.1 (1.9)	<0.001	2.7 (1.5)	4.3 (1.7)	<0.001
**LN diameter in PET/CT (cm)**						
Mean (SD)	0.4 (0.4)	1.0 (0.7)	<0.001	0.3 (0.4)	1.2 (1.1)	<0.001
**tSUVmax**						
Mean (SD)	8.3 (6.1)	11.2 (6.3)	<0.001	8.6 (6.3)	11.7 (5.1)	<0.001
**nSUVmax**						
Mean (SD)	0.7 (1.1)	4.5 (4.5)	<0.001	0.6 (1.1)	4.6 (5.4)	<0.001
**nSUVmax/tSUVmax**						
Mean (SD)	0.1 (0.2)	0.5 (0.5)	<0.001	0.1 (0.2)	0.4 (0.6)	<0.001
Uterine corpus invasion in PET/CT, n (%)			<0.001			<0.001
No	163 (94.2)	34 (55.7)		114 (87.0)	27 (45.0)	
Yes	10 (5.8)	27 (44.3)		17 (13.0)	33 (55.0)	
LN status in PET/CT, n (%)			<0.001			<0.001
No	132 (76.3)	12 (19.7)		105 (80.2)	18 (30.0)	
Yes	41 (23.7)	49 (80.3)		26 (19.8)	42 (70.0)	

*LN, lymph node; SCCA, squamous cell carcinoma associated antigen; SUVmax, maximum standardized uptake value; nSUVmax, SUVmax of lymph node; tSUVmax, SUVmax of tumor; PET/CT, positron emission tomography/computed tomography.*

**FIGURE 1:**
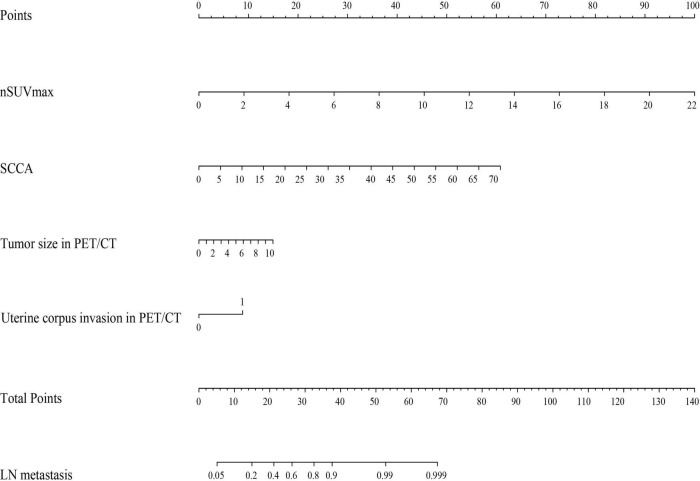
A nomogram predicting the risk of lymph node metastasis in early-stage cervical cancer patients. nSUVmax, maximum standardized uptake value of lymph node; SCCA, squamous cell carcinoma associated antigen; PET/CT, positron emission tomography/computed tomography; LN, lymph node.

According to the results of our final analysis in primary cohort (Table 2), SCCA (OR: 1.134; 95% CI: 1.005–1.278; *P* = 0.041), nSUVmax (OR: 1.890; 95% CI: 1.075–3.323; *P* = 0.027), uterine corpus invasion in PET/CT (OR: 4.229; 95% CI: 1.269–14.092; *P* = 0.019), and tumor size in PET/CT (OR: 1.420; 95% CI: 1.049–1.921; *P* = 0.023) are independent hazardous factors for lymph node metastasis in patients with cervical cancer. Ultimately, SCCA, nSUVmax, uterine corpus invasion, and tumor size in PET/CT were chosen to construct a nomogram to predict the lymph node metastasis in cervical cancer patients (Figure 1).

**TABLE 2:** The variables identified by logistic multivariable regression analysis.

Variable	Multivariable analysis		
	OR	95% CI	*P*
Age (year)			0.520
<50	Reference		
≥50	1.662	0.353–7.827	
Menopause			0.300
No	Reference		
Yes	0.444	0.096–2.064	
SCCA (ng/mL)	1.134	1.005–1.278	0.041
Tumor size in PET/CT (cm)	1.420	1.049–1.921	0.023
LN diameter in PET/CT (cm)	1.183	0.360–3.886	0.781
tSUVmax	0.950	0.850–1.062	0.366
nSUVmax	1.890	1.075–3.323	0.027
nSUVmax/tSUVmax	1.725	0.051–58.313	0.762
Uterine corpus invasion in PET/CT			0.019
No	Reference		
Yes	4.229	1.269–14.092	

*OR, odds ratio; CI, confidence interval.*

**FIGURE 2:**
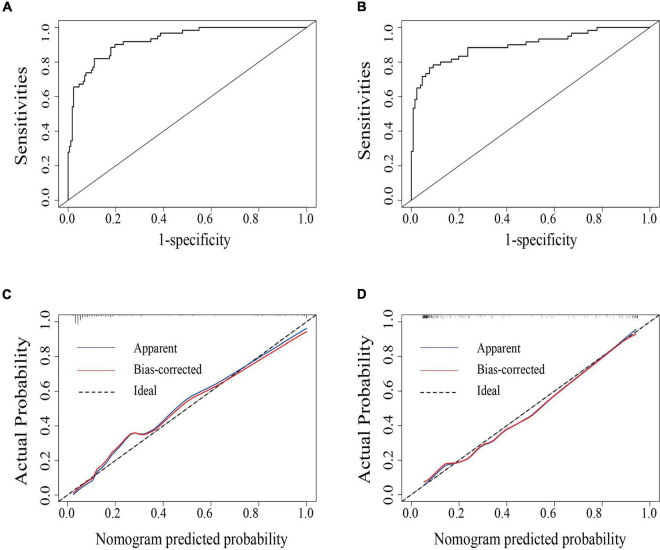
The area under the receiver operating characteristics curve (AUC-ROC) of the model in the primary **(A)** and the validation cohort **(B)**, respectively. Calibration curves of the nomogram for the primary **(C)** and validation cohort **(D)**.

**FIGURE 3:**
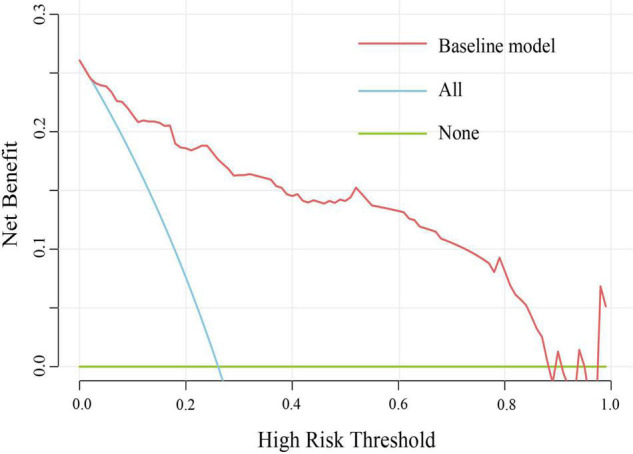
Decision curve analysis for the nomogram.

The area under the receiver operating characteristic curve was 0.926 (95% CI: 0.890–0.962) in the primary cohort and was 0.897 (95% CI: 0.843–0.951) in the validation cohort, indicating that the nomogram has robust discrimination (Figures 2A,B). And calibration curves of the nomogram showed that the excellent concordance between the probability predicted by the nomogram and the actual probability in both groups (Figures 2C,D). The decision curve showed that if the threshold probability of a patient is between 5 and 90%, using the nomogram to predict lymph node metastasis can add more benefit than either the treat-all or treat-none scheme (Figure 3).

The sensitivity, specificity, PPV, NPV, and accuracy of PET/CT for LN metastasis were 75.2, 78.0, 57.6, 88.8, and 77.2, respectively. Compared with PET/CT alone, our nomogram showed risen sensitivity (82.0, 78.3%), specificity (89.0 and 90.8%), PPV (72.5 and 79.7%), NPV (93.3 and 90.2%), and accuracy (87.2 and 86.9%) in both the primary and validation cohorts (Table 3).

**TABLE 3:** Accuracy values of PET/CT and nomogram in LN metastasis.

Variable	PET/CT	Nomogram	
		Training cohort	Validation cohort
Sensitivity	75.2%	82.0%	78.3%
Specificity	78.0%	89.0%	90.8%
Positive predictive value (PPV)	57.6%	72.5%	79.7%
Negative predictive value (NPV)	88.8%	93.3%	90.2%
Accuracy	77.2%	87.2%	86.9%

*PET/CT, positron emission tomography/computed tomography; LN, lymph node.*

## Discussion

In 2018, the presence of lymphatic involvement is included in FIGO staging system because of the prognostic importance of lymphatic involvement. In addition, except for the clinical examination, imaging and pathologic findings could be used to determine the stage of the disease. Thus, the precise evaluation of lymph node is very key to guide the treatment and adjuvant radiotherapy postoperatively. In the present study, based on the univariate and multivariate analysis, we identified four factors that were associated with prediction of lymph node metastasis: SCCA, nSUVmax, uterine corpus invasion, and tumor size in PET/CT. Then, we constructed a nomogram to predict the risk of nodal metastasis in early-stage CC. The model presented high specificity and sensitivity.

In recent years, MRI is increasingly applied for the pre-treatment evaluation of local spread of cervical cancer, on the basis of its excellent soft-tissue contrast and high spatial resolution. Radiomics involves the process of the conversion of medical images into high-dimensional, mineable data *via* the automated high-throughput extraction of quantitative imaging features. Hou et al. developed and validated a multiparametric MRI-based radiomics model to evaluate the tumor size, local invasive and LN status in patients with CC (11). Similarly, Xiao et al. constructed a nomogram that incorporates the radiomics signature, MRI-reported LN status, and FIGO stage to predict LN status in patients with early-stage CC (12). Although these studies based on MRI parameters showed favorable discriminative ability for tumor size and local invasive, it presents limitation for evaluating the status of LN metastasis. ^18^F-FDG PET/CT is a functional method based on the increased glucose metabolism of cancer cells, and can often detect tiny metastatic lymph nodes ranging in size from 5 to 9 mm (13). PET/CT has a unique role in differentiating benign and malignant tumors, evaluating the efficacy of radiotherapy and chemotherapy and the prediction of tumor metastasis, etc. (14, 15). Previous studies had shown that PET/CT was superior to CT and MRI in the evaluation of retroperitoneal lymph nodes, whose sensitivity and specificity was 48.6–82% and 75–98%, respectively (6, 7, 16). However, PET/CT also has some shortcomings. Some benign lymph node diseases can also display high levels of ^18^F-FDG uptake, making it misdiagnosed as a metastatic lymph node and increasing the false positive rate (17). Therefore, many studies aiming to improve the accurate of PET/CT was performed. For example, Wang et al. developed a nomogram to predict para-aortic lymph node (PALN) involvement. However, all stages of cervical cancer were included. PALN was only assessed by imaging (PET/CT or CT). As previously stated, assessment of node involvement by imaging is far from perfect. A Korean team developed a score based on tumor size and PALN involvement on PET/CT with patient who underwent para-aortic lymphadenectomy in locally advanced cervical cancer (LACC). Likewise, the main limitation of this study is that patients with LACC did not undergo surgical staging. These two studies may have under-estimated the FIGO stage and may have included patients with an initially advanced cervical cancer. Recently, Liu et al. constructed a non-invasive and convenient nomogram based on two parameters (SCCA and nSUVmax) for preoperative evaluation of pelvic lymph node metastasis in early-stage CC and confirmed it had good diagnostic ability (18–20). However, this study focused on only quantitative parameters of PET/CT (SUVmax), ignoring the effect of tumor size and the uterine corpus invasion.

In early staging systems, patients with a lesion confined to the cervix but extending to the endometrium were regarded as Stage II. However, uterine corpus invasion was disregarded over time. In the 2018 FIGO cervical cancer staging system, uterine corpus invasion has also been disregarded as in previous FIGO staging systems. According to this stage system, involvement of adjacent anatomic structures is associated with a worse prognosis and alters the FIGO stage, except uterine corpus invasion (9). However, previous studies have shown uterine corpus invasion was an independent risk factor for the prognosis of early cervical cancer patients and verified that it had an association to pelvic lymph node metastasis in CC (21). Hope et al. demonstrated that uterine corpus invasion of cervical cancer was correlated with the presence of lymph node metastasis in PET/CT (22). Uterine corpus invasion in MRI was independently associated with lymph node metastasis (23). It is known that para-aortic/pelvic nodal metastasis is very important in the decision for the need for adjuvant radiotherapy, the need for para-aortic lymphadenectomy or the border of the radiotherapy field. By evaluating the uterine invasion, it is easy to predicate the para-aortic/pelvic nodal metastasis. Another predictive factor of our nomogram was tumor size in PET/CT. Evaluation by PET/CT is part of the standard local-regional spread assessment for CC. Various studies have demonstrated that tumor size is an independent prognostic factor in determining the stage of CC. Togami et al. showed that tumor size greater than 2 cm was independently associated with LNM. Similarly, Han et al. proven tumor size > 3.5 cm was connected with para-aortic lymph node metastasis. This was confirmed by Kim et al., who found that a larger tumor size assessed by MRI was an independent predictor of nodal metastases (8, 24–26). In the present study, our univariate and multivariate analysis also confirmed that uterine corpus invasion and tumor size in PET/CT were associated with high risk of LN metastasis in CC. This may explain the high rate of distant metastasis in endothelial cancer or the presence of uterine invasion of CC in patients treated with radiotherapy (27). Our study confirmed that tumor histology was not associated with LNM, which was consistent with previous reports (8). Recently, a large-scale retrospective study also demonstrated that histological type was not an independent risk factor for LNM in cervical cancer (28). Thus, our nomogram included the corpus invasion and tumor size in PET/CT. When we constructed the nomogram based the four factors, we found our nomogram had higher sensitivity (82.0%, 78.3% vs. 70.5%, 73.1%) and NPV (93.3%, 90.2% vs. 72.3%, 73.1%) and accuracy (87.2%, 86.9% vs. 81.3%, 79.2%) than previous, whether in the primary cohort or the validation cohort (6). Meanwhile, the calibration curve and DCA showed that the nomogram has good clinical applicability.

Our study still had some limitations. First of all, although internal and external verification certificated the model fits well, the retrospective nature may therefore introduce the possibility of some inevitable recall bias. Prospective studies are still required to confirm the predictive value of the model in a clinical practice environment. In addition, in spite of all patients accepting pelvic lymph node dissection, not all underwent para-aortic lymph node dissection, and patients who did not undergo para-aortic lymph node dissection were regarded their para-aortic lymph nodes negative. In fact, lymph node metastasis has always been considered a gradual process in cervical cancer. However, some studies reported that 25–31.2% CC patients had skip lymph node metastases. The latest multiple prospective studies had certified that the rate of skip para-aortic lymph node metastases in cervical cancer was 3.3–6.0% (29–31).

In conclusion, we have established and validated an effective nomogram for predicting preoperative LN metastasis in early-stage cervical cancer. This individualized model provides a more effective and non-invasive preoperative means of assessing patients risk of LN metastasis. The model may identify a low-risk group of patients who would least benefit from a surgical treatment and give a further guideline in the decision for the need for appropriate adjuvant treatment after surgery.

## Data Availability Statement

The raw data supporting the conclusions of this article will be made available by the authors, without undue reservation.

## Ethics Statement

The studies involving human participants were reviewed and approved by the Ethics Committee of Obstetrics and Gynecology Hospital, Fudan University, Shanghai, China (No. 2020-183). The patients/participants provided their written informed consent to participate in this study.

## Author Contributions

KH: protocol/project development. CBL: manuscript editing. SY and CLL: manuscript writing, data analysis, and data collection or management. All authors contributed to the article and approved the submitted version.

## Conflict of Interest

The authors declare that the research was conducted in the absence of any commercial or financial relationships that could be construed as a potential conflict of interest.

## Publisher’s Note

All claims expressed in this article are solely those of the authors and do not necessarily represent those of their affiliated organizations, or those of the publisher, the editors and the reviewers. Any product that may be evaluated in this article, or claim that may be made by its manufacturer, is not guaranteed or endorsed by the publisher.

## Footnotes

1
http://www.r-project.org
